# Mitigating the Vanishing Gradient Problem Using a Pseudo-Normalizing Method

**DOI:** 10.3390/e28010057

**Published:** 2025-12-31

**Authors:** Yun Bu, Wenbo Jiang, Gang Lu, Qiang Zhang

**Affiliations:** 1 School of Electrical Engineering and Electronic Information, Xihua University, Chengdu 610039, China; jiangwenbo@mail.xhu.edu.cn (W.J.); ganglu@mail.xhu.edu.cn (G.L.); 2 School of Sciences, Southwest Petroleum University, Chengdu 610500, China; zhang@swpu.edu.cn

**Keywords:** deep learning, convolutional neural networks, backpropagation algorithm, vanishing gradient, pseudo-normalizing

## Abstract

When training a neural network, the choice of activation function can greatly impact its performance. A function with a larger derivative may cause the coefficients of the latter layers to deviate further from the calculated direction, making deep learning more difficult to train. However, an activation function with a derivative amplitude of less than one can result in the problem of a vanishing gradient. To overcome this drawback, we propose the application of pseudo-normalization to enlarge some gradients by dividing them by the root mean square. This amplification is performed every few layers to ensure that the amplitudes are larger than one, thus avoiding the condition of vanishing gradient and preventing gradient explosion. We successfully applied this approach to several deep learning networks with hyperbolic tangent activation for image classifications. To gain a deeper understanding of the algorithm, we employed interpretability techniques to examine the network’s prediction outcomes. We discovered that, in contrast to popular networks that learn picture characteristics, the networks primarily employ the contour information of images for categorization. This suggests that our technique can be utilized in addition to other widely used algorithms.

## 1. Introduction

Conventional neural networks typically have a limited number of layers because of the problem of vanishing gradients. Such networks frequently utilize activation functions with a derivative of less than one, such as logistic sigmoid or hyperbolic tangent (tanh), which is assumed to primarily result in the vanishing gradient problem. As such, researchers have developed techniques to successfully train deeper and larger neural networks. For example, the deep belief network is an early method that uses unsupervised pre-training and supervised fine tuning [1]. However, owing to its complexity in comparison to the stochastic gradient descent (SGD)-based methods, this approach is inefficient for learning.

Glorot and Bengio [2] proposed a normalized initialization strategy for approximately maintaining variances of backpropagated gradients when they cross layers during training.

Considerable advancements in deep learning over the past 10 years have been driven by the adoption of rectifier activation function units (ReLUs) [3], batch normalization (BN) [4], and residual networks [5]. ReLU, which is defined as f(x)=max(0,x), has been extensively utilized because of its simplicity and high computational efficiency, allowing the deep sparse rectifier neural network to outperform the tanh-based network [3]. Currently, neural networks have become deeper as the utilization of ReLU can only partly overcome the problem of vanishing gradients.

A main merit of ReLU is that if each layer’s input data can be carefully arranged to ensure some data are larger than zero, the gradient can be propagated backwards efficiently. However, in a deep network, the change in the distribution of the input data could be exaggerated, after which BN can be introduced before the activation to white inputs with a mean of zero and a variance of one [4]. Essentially, the vanishing gradient problem can be addressed as described earlier, and any activation function can be applied if BN is contained, yet the byproduct is the higher computation overhead.

However, with the increase in the depth of a neural network, its performance degrades. Therefore, He et al. [5] proposed a residual network, with a shortcut from input to output to fit an identity mapping. In addition, the network was able to address the problem of vanishing gradients. Since then, residual learning gained popularity in deep learning.

With significant advancements, current deep learning networks possess hundreds of layers and are employed in various areas [6,7,8,9,10,11]. Studies have also proposed and designed new neural networks with deeper, more complex structures, as well as new algorithms and new records [12,13,14,15,16,17,18]. However, the training of most deep learning networks is still difficult [19], and several studies have put forward valuable views and novel solutions [2,20,21] to address the vanishing gradient problem. To comprehend the influence of various activation functions on model performance, our initial focus is to analyze whether updates in network parameters during the training process will steer the model’s evolution in the anticipated direction.

For a neural network, the *l*-th input and output expression is defined as follows:

(1) $$\begin{array}{l} o^{\left(l\right)}=w^{\left(l\right)}a^{\left(l-1\right)}+b^{\left(l\right)}\\ a^{\left(l\right)}=f(o^{\left(l\right)})=f(w^{\left(l\right)}a^{\left(l-1\right)}+b^{\left(l\right)}) \end{array}$$

where *l* denotes the *l*-th layer, $w^{\left(l\right)}$ denotes the parameter matrix, $o^{\left(l\right)}$ is its linear output, f(.) is the activation function, and $a^{\left(l\right)}$ is the *l*-th layers’ output vector.

For simplification, considering a simple two-layer neural network without bias, each layer’s output is calculated as follows:

(2) $$a^{\left(1\right)}=f(w^{\left(1\right)}x),\;a^{\left(2\right)}=f(w^{\left(2\right)}a^{\left(1\right)})$$

where x is the input data of the network.

During learning process, after weights are updated, the network will perform a new feedforward. The new outputs and their first-order approximations are computed using (3), because ${\Delta w}^{\left(1\right)}$ and $∆w^{\left(2\right)}$ are computed based on x, $a^{\left(1\right)}$, and $a^{\left(2\right)}$.

(3) $$\begin{array}{cc} a_{new}^{\left(1\right)} & =f((w^{\left(1\right)}+\Delta w^{\left(1\right)})x)\dot{=}a^{\left(1\right)}+f^{\prime }(w^{\left(1\right)}x)\cdot \Delta w^{\left(1\right)}x\\ a_{new}^{\left(2\right)} & =f((w^{\left(2\right)}+\Delta w^{\left(2\right)})a^{\left(1\right)})\\ & ≐f(w^{\left(2\right)}a^{\left(1\right)})+f^{\prime }(w^{\left(2\right)}a^{\left(1\right)})\cdot \Delta w^{\left(2\right)}a^{\left(1\right)} \end{array}$$

However, the actual network output can be approximated as

(4) $$\begin{array}{cc} a_{new\_true}^{\left(2\right)} & =f((w^{\left(2\right)}+\Delta w^{\left(2\right)})a_{new}^{\left(1\right)})\\ & \approx f(w^{\left(2\right)}a_{new}^{\left(1\right)})+f^{\prime }(w^{\left(2\right)}a_{new}^{\left(1\right)})\cdot \Delta w^{\left(2\right)}a_{new}^{\left(1\right)}\\ & =f(w^{\left(2\right)}a_{new}^{\left(1\right)})+f^{\prime }(w^{\left(2\right)}a_{new}^{\left(1\right)})\cdot \Delta w^{\left(2\right)}(a^{\left(1\right)}+f^{\prime }(w^{\left(1\right)}x)\cdot \Delta w^{\left(1\right)}x) \end{array}$$

Since the gradient of each layer is computed under the assumption that the coefficients of all layers are fixed, a comparison of Equations (3) and (4) reveals that changes in the coefficients of the preceding layer cause the updates in the subsequent layer’s coefficients to deviate from the originally calculated direction. Considering only the items of the second components $\Delta w^{\left(2\right)}a^{\left(1\right)}$ and $∆w^{\left(2\right)}{(a}^{\left(1\right)}+f^{\prime }\left(w^{\left(1\right)}x\right)∆w^{\left(1\right)}x)$, the first layer’s derivative, $f^{\prime }\left(w^{\left(1\right)}x\right)$, is an important factor that could result in weight shifting. A deep learning network with smaller derivative activation functions may be more easily trained and more robust with an increment in noise or if the input data is polluted by noise.

In addition, there are several application scenarios with smaller data scales, such as function fitting, which are more suitable for a compact neural network than a deeper one. Therefore, we reconsidered the use of smoother, sigmoid-like activation functions in deep learning, albeit with a higher calculation cost than that of ReLU. This can be solved by combining BN or Resnet and sigmoid-like functions; however, this method may increase the training overhead. A deep network with saturated activation functions is prone to vanishing gradients because its derivatives within [0, 1] will multiply sequentially. To overcome this drawback, some gradients could be enlarged every few layers. This strategy can mitigate the vanishing gradient condition, thus achieving the goal of this study.

## 2. Related Research

As mentioned, the most important methods that can overcome the vanishing gradient problem involve the use of ReLU, BN, and residual networks. In this section, we analyze the merits and shortcomings of using these components in designing a neural network.

### 2.1. ReLU Activation

ReLU and its modifications are the most used activations because of their merits, such as no gradient vanishing, simple expression, and low calculation costs. Other advantages of ReLUs are as follows: (i) They omit or suppress neurons less than zero, which ensures that each layer outputs less; this ensures the network can filter meaningless information or some noise. In other words, the network possesses a low level of attention ability. (ii) The omission of some neurons achieves a similar effect as a dropout [3], which inactivates some neurons randomly. This is very helpful against model overfitting. (iii) The process of activation allows equal treatment of neurons larger than zero, demonstrating detail-preserving properties superior to sigmoid-like functions, which can suppress values farther from zero, thus resulting in an indistinctive output.

Nevertheless, ReLU also has some drawbacks. For example, ReLU yields asymmetric outputs that can cause hide layers output distribution shifting and are no longer whitened, defined as internal covariate shift [4], which is an obstacle in training. Second, the positive output range of ReLUs is unlimited and, coupled with softmax and cross-entropy loss during classification, the absolute weights would be forced to grow, resulting in the risk of gradient explosion. To overcome this problem, regularization and clipping operations must be considered during learning.

### 2.2. Batch Normalization (BN)

BN was proposed [4] to fix the distributions of the input layer and avoid the saturated regime to prevent the gradient from disappearing into feedback propagation. BN is now commonly used in deep learning to accelerate network convergence. In training, the inputs are whitened using the estimated mean and variance of each batch; then, the normalized values are linearly transformed as the outputs of BN.

Although BN demonstrates faster convergence and lower dropout rates, it has many drawbacks. For instance, a BN operation can result in a higher computation cost because the mean and variance of each batch must be calculated, in turn slowing down the training process.

Another drawback of BN is that it introduces a dependency on the batch size during training. That is, the statistics computed during training, such as the mean and variance of a batch, may not accurately represent the true population statistics. Therefore, when the batch size used during testing or inference differs from that used during training, the model performance may be affected. Additionally, BN may not work well for small batch sizes, as the estimated mean and variance in such cases may not be reliable, leading to unstable training and degraded performance.

### 2.3. Deep Residual Learning

Network performance is known to degrade if more layers are added to a neural network. According to Equations (3) and (4), with an increase in the depth, the network may not be updated toward our expected direction by error backpropagation. A larger number of layers complicate the deliberation of performance. Therefore, deep networks cannot easily and naturally learn useful features as was expected.

To address this issue, researchers propose the use of a residual network (ResNet). The ResNet introduces shortcuts or skip connections, which help alleviate the vanishing gradient problem to some extent and play a crucial role in transporting original or lower-level information to higher layers, ensuring similar properties for the input and output layers. The original features ensure that the hidden layers do not output much meaningless information. Consequently, ResNet surpasses almost all the existing deep learning structures, making it the cornerstone of the most state-of-the-art backbones.

However, ResNet and its variants with many shortcut connections can complicate network structures; whether some connections among them are redundant is not clear. More matrices and memory are needed to adjust the outputs of activations to adapt to the size of the tensors. Shortcut connections are primarily designed to address the challenges attributed to training very deep networks. However, for small-scale data applications or data compression [22], the benefits of shortcut connections may not be as significant, and they may not provide substantial performance improvement.

## 3. Pseudo-Normalizing Layer Gradients (PN)

### 3.1. Revising the Backpropagation Algorithm

The goal of training a neural network is to find the optimal weights that can minimize a cost function C(w). Then, the heart of using the feedback update algorithm is to calculate the partial derivative ∂C/∂w of each layer using the following expressions:

(5) $$\begin{array}{l} \delta^{\left(l\right)}=\frac{\partial C}{\partial a^{\left(l\right)}}⊙f^{\prime }(o^{\left(l\right)})\\ =\left\{\begin{array}{l} \frac{\partial C}{\partial a^{\left(L\right)}}⊙f^{\prime }(o^{\left(L\right)})\;\;l=L,\;\mathrm{at}\;\mathrm{output}\;\mathrm{layer}\\ {\left(w^{\left(l+1\right)}\right)}^{T}\delta^{\left(l+1\right)}⊙f^{\prime }(o^{\left(l\right)})\;\;l<L,\;\mathrm{at}\;l-th\;\mathrm{layer} \end{array}\right. \end{array}$$

(6) $$\frac{\partial C}{\partial w^{\left(l\right)}}=a^{\left(l-1\right)}\delta^{\left(l\right)}$$

where ⊙ is the Hadamard product, $({w^{\left(l+1\right)})}^{T}$ is the transpose of the weight matrix, and *L* is the total number of layers.

The famous vanishing/exploding problem of gradients arises in backpropagation-based training. To simply demonstrate it, we suppose that each layer’s weight is a scale and, using the chain rule, we can achieve the partial derivative of the *l*-th layer:

(7) $$\begin{array}{c} \frac{\partial C}{\partial w^{\left(l\right)}}=\frac{\partial C}{\partial a^{\left(L\right)}}f^{\prime }(o^{\left(L\right)})w^{\left(L\right)}f^{\prime }(o^{\left(L-1\right)})\times \\ \cdots w^{\left(l+1\right)}f^{\prime }(o^{\left(l\right)})a^{\left(l-1\right)} \end{array}$$

Then, Equation (7) can be streamlined further by omitting the weights and obtaining Equation (8).

(8) $$\frac{\partial C}{\partial w^{\left(l\right)}}=\frac{\partial C}{\partial a^{\left(L\right)}}f^{\prime }(o^{\left(L\right)})f^{\prime }(o^{\left(L-1\right)})\cdots f^{\prime }(o^{\left(l\right)})a^{\left(l-1\right)}$$

Equations (7) and (8) illustrate that the weights and partial derivatives of all the higher layers contribute to the gradients of the lower layers; however, the vanishing/exploding gradient is mainly caused by the latter. For example, a net with sigmoid and tanh activations will experience vanishing gradients because they have derivatives that are smaller than a unit magnitude. In addition, backpropagated gradients decrease in size with increasing distance from the output layer toward the input layer.

However, if not all $\left|f\prime (o^{\left(l\right)})\right|$ in (8) are smaller than 1, the vanishing gradient problem may be relieved. This is the motivation of our algorithm—that the interval enlarges some derivatives to ensure that gradients are large enough to cross all layers.

### 3.2. The Theoretical Basis of the Proposed Algorithm

Assuming two independent random variables, x and y, follow the Gaussian distributions:

(9) $$x∼N(0,\;\sigma_{x}^{2})\;\;y∼N(0,\;\sigma_{y}^{2})$$

where we assume they are of 0 means and standard deviations, $\sigma_{x}$ and $\sigma_{y}$, respectively.

Their product also follows a new Gaussian distribution:
  (10) $$xy∼N(0,\;\frac{\sigma_{x}^{2}\sigma_{y}^{2}}{\sigma_{x}^{2}+\sigma_{y}^{2}})$$
  When only one variance is normalized, for example, $\sigma_{x}^{2}=1$, then
  (11) $$xy∼N(0,\;\frac{\sigma_{y}^{2}}{1+\sigma_{y}^{2}})$$
  If $\sigma_{y}^{2}\ll 1$, then $\frac{\sigma_{y}^{2}}{1+\sigma_{y}^{2}}\approx \sigma_{y}^{2}$.

The equations reveal that when multiplying two Gaussian signals, if one of them is normalized, the variance of the resulting signal is amplified but only becomes equivalent to the unnormalized one. By applying this method to normalize the derivatives of Equation (8), the vanishing problem can be alleviated.

Some popular activation functions, like sigmoid and tanh, are of finite derivatives within (0, 1). They are not symmetrical distributions, but they can be converted into a symmetrical distribution’s random signal.

Assuming x is a random sequence greater than 0 with a limited range, another random sequence t can be generated from x according to the following equation:

(12) $$t(k)={\left(-1\right)}^{n}x(k)$$

where *n* is a random number that is either 0 or 1 with a 50% probability.

The new random sequence *t* is symmetrically distributed; its mean is E(t)=0, and its variance is

(13) $$E(t^{2})=\sigma_{t}^{2}=E(x^{2})$$

where function E(.) represents the mean value of a random variable.

If the sequence t is close to the Gaussian distribution, Equation (11) can also be suitable for x, and another advantage of the processing is that it can also omit computing and using the average value to obtain the variance.

### 3.3. Amplifying Part Layers’ Derivatives Through Pseudo-Normalization

To break the conditions causing gradient disappearance, the proposed algorithm enlarges some layers’ derivatives using the pseudo-normalizing operators defined as

(14) $$\sigma^{\left(l\right)}=\sqrt{\frac{1}{m}\sum_{i=1}^{m}{\left(f^{\prime }(o_{i}^{\left(l\right)})\right)}^{2}},$$

(15) $$norm(f^{\prime }(o^{\left(l\right)}))=\frac{f^{\prime }(o^{\left(l\right)})}{\sigma^{\left(l\right)}},$$

where m is the total number of outputs in the *l*-th layer, σ denotes the root mean square, and *norm*(x) is the pseudo-normalization (PN) function; the name comes from the fact that it does not subtract the mean.

However, it is important to note that if the derivatives are Gaussian distributed, approximately 32% of the normalized elements may exceed 1, which could lead to a gradient explosion if all the derivatives are normalized in (8). Therefore, a feasible solution is to selectively amplify some of the derivatives. Choosing which layer derivative to amplify will not affect the performance of the model. In the actual training, for simplicity, the derivatives of every other layer will be magnified.

(16) $$\begin{array}{l} \frac{\partial C}{\partial w^{\left(l\right)}}\propto \frac{\partial C}{\partial a^{L}}f^{\prime }(o^{\left(L\right)})norm(f^{\prime }(o^{\left(L-1\right)}))\times \\ f^{\prime }(o^{\left(L-2\right)})norm(f^{\prime }(o^{\left(L-3\right)}))\cdots a^{\left(l-1\right)} \end{array}$$

In the expression, the first term $\frac{\partial C}{\partial a^{L}}$ is dominant; larger costs will cause larger update amounts of all weights, and vice versa.

This expression is only used as a simple illustration of our algorithm, wherein the key item is the stacked combination derivate, $norm(f^{\prime }(o^{\left(l\right)})f^{\prime }(o^{\left(l-1\right)})$, which guarantees that gradients will not disappear quickly in the flowback. However, if a network is very deep, according to (11), gradients will still vanish. Fortunately, normalizing convolutional layers will result in larger gradients than dense layers, which will enable gradients to effectively transfer to more layers.

### 3.4. Convolution Layers Generate Larger Gradients than Dense Layers

The convolution layer is one of the basic components of a deep learning network; compared to a dense layer, it has a larger partial derivative and error. According to the chain rule, the error and gradients of a convolutional layer can be calculated using the equation

(17) $$\frac{\partial C}{\partial w^{\left(l\right)}}=a^{\left(l-1\right)}*\delta^{\left(l\right)}\delta^{\left(l-1\right)}=\delta^{\left(l\right)}*(rot180(w^{\left(l\right)})⊙f^{\prime }(o^{\left(l-1\right)}))$$

where ∗ is a convolution operator and rot180(·) denotes rotating 180 degrees: the coefficient matrix is first flipped horizontally and then flipped vertically.

If the output feature map shape is [fw, fh], each rate of change of weights will be the sum of the fw × fh product of the input image and feedback error. This results in the gradient of a convolutional layer being larger than that of a dense layer. Similarly, the error fed back to the next layer will also be larger. Therefore, if the range of $f^{\prime }(o^{\left(l\right)})$ is enlarged, according (17), the impact of the amplified error $\delta^{\left(l-1\right)}$ will spread to further layers, not just one layer as Equation (11). This also implies that gradient clipping should be included in the training process to avoid gradient explosion.

### 3.5. The Proposed Backpropagation Algorithm

The stacking of combination derivates can increase some derivates to larger than 1, ensuring that the backpropagated gradient is large enough. According to Equation (14), half the layers’ partial derivates will be enlarged. However, this could result in higher computation costs. In addition, as mentioned earlier, a larger derivate will result in more shifts in the next layers. Therefore, we propose a training strategy with computation simplicity.

**Figure 1:**
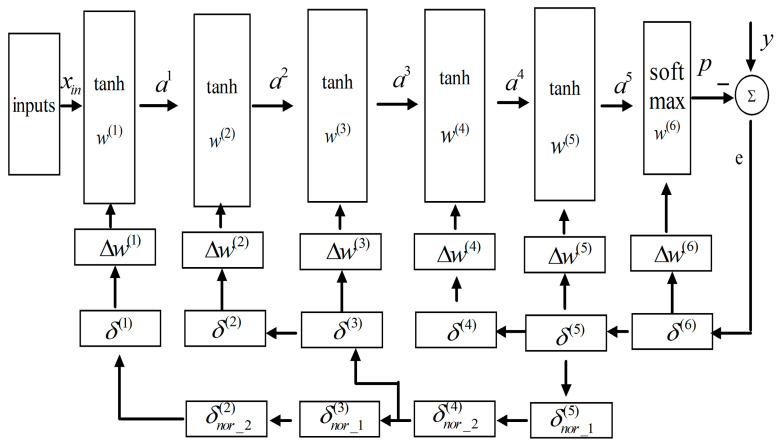
Proposed backpropagation learning algorithm.

To describe the method clearly, we designed a neural network with six layers for classification. Each layer, except the top softmax layer, uses tanh as the nonlinearity function and cross entropy as the loss. The complete training method is shown in Figure 1.

Figure 1 shows that except for the output and input, other errors are calculated twice for two different feedback paths, such as $\delta^{\left(5\right)}$ and $\delta_{nor\_1}^{\left(5\right)}$; the former is passed through limited layers to work out the $\Delta w^{\left(l\right)}$ and/or the next layer’s $\delta^{\left(l\right)}$, while the latter can be transported through all remaining layers to compute the errors defined in Equation (18). As shown, the first feedback demonstrates the errors in the top layers from *δ*^(6)^ to *δ*^(4)^ or from *δ*^(3)^ to *δ*^(2)^. The second feedback demonstrates bottom propagation, wherein the lower errors are calculated by passing back combination errors. The cascading combination errors with distinct subscripts are defined as

(18) $$\begin{array}{l} \delta_{nor\_1}^{\left(l\right)}={\left(w^{\left(l+1\right)}\right)}^{T}\delta^{\left(l+1\right)}⊙norm(f^{\prime }(o^{\left(l\right)}))\\ \delta_{nor\_2}^{\left(l-1\right)}={\left(w^{\left(l\right)}\right)}^{T}\delta_{nor\_1}^{\left(l\right)}⊙f^{\prime }(o^{\left(l-1\right)}) \end{array}$$

where $\delta_{nor\_1}^{\left(l\right)}$ and $\delta_{nor\_2}^{\left(l-1\right)}$ are the combination derivate. According to Figure 1, the next two errors can be calculated as

(19) $$\begin{array}{l} \delta^{\left(3\right)}=({\left(w^{\left(4\right)}\right)}^{T}\delta_{nor\_2}^{\left(4\right)}⊙f^{\prime }(o^{\left(3\right)})\\ \delta^{\left(2\right)}=({\left(w^{\left(3\right)}\right)}^{T}\delta^{\left(3\right)}⊙f^{\prime }(o^{\left(2\right)}) \end{array}$$

In this way, all gradients can be computed, and a combination derivate is utilized to compute two errors because a conventional neural network has two or three layers to avoid gradient vanishing. If the propagated gradient is not very small, more errors can be calculated by one combination error. For example, we can obtain $\delta^{\left(1\right)}$ through $\delta_{nor\_1}^{\left(5\right)}\to \delta_{nor\_2}^{\left(4\right)}\to \delta^{\left(3\right)}\to \delta^{\left(2\right)}\to \delta^{\left(1\right)}$. Therefore, the training process is simplified. In addition, the second feedback initiates from $\delta_{nor\_1}^{\left(5\right)}$ and not from the output, because the gradient of cross-entropy coupled with the softmax output is sufficiently large.

## 4. Experiments

To verify the feasibility of the proposed algorithm, we designed a 10-layer network with tanh activation; the network comprises several convolutional neural layers followed by some fully connected layers. The network was used for the classification of CIFAR-10, CIFAR-100, and the Real Doppler RAD-DAR (RDRD) datasets [23,24]. Notably, as analyzed earlier, most of the deep learning networks that can achieve state-of-the-art performance are based on a skip-connection structure, and they are not significantly affected by gradient vanishing. Therefore, our algorithm is not suitable for these structures. The models in simulations are only used to check the effectiveness of the proposed algorithm.

Although the learning rate is generally considered the most important hyper-parameter [25,26], the optimal parameter is often unknown; therefore, we used the cosine learning strategy [27] in training.

The LeCun normal was used as the weight initialization scheme. It is defined as a zero-mean Gaussian distribution with a standard deviation of $1/\sqrt{f_{in}}$, where $f_{in}$ is the input neuron number. All images including the test dataset are normalized as

(20) $$\hat{x}=\frac{x-E(x)}{\sqrt{Var(x)}}$$

In this study, we used the SGD algorithm and set the mini-batch size to 50. To avoid gradient explosion in experiments, we also set up the gradient clipping and limited the gradient to between −0.5 and 0.5.

### 4.1. CIFAR-10 Classification

CIFAR-10 dataset is a popular benchmark that comprises ten classes with 50,000 pictures for training and 10,000 for testing. Two networks with the 10 used network layers were designed, of which model 1 comprised 8 convolutional layers, 2 fully connected layers, with tanh activation, as listed in Table 1. Model 2 had 4 convolutional layers and 6 dengsens.

We trained model 1 for 90 epochs; in the last 20 epochs, the learning rate was fixed to a value, i.e., 0.0001, and the regularization was a constant value, 0.003, during training.

**Figure 2:**
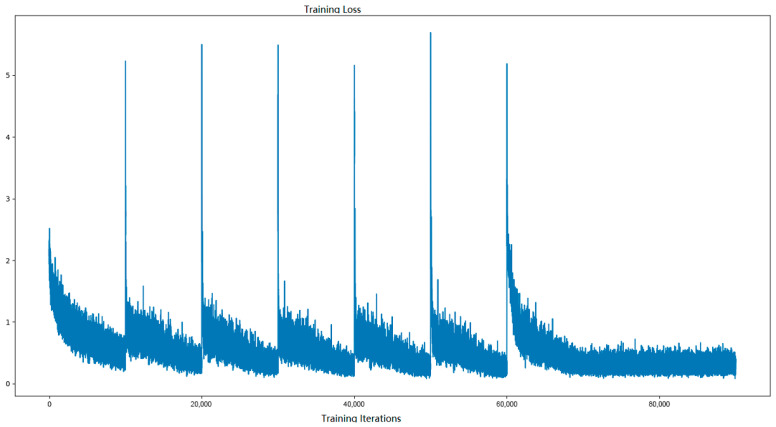
Training loss.

Figure 2 shows the training loss in the declining period of the learning rate. In particular, in the first cycle of training, the loss curve drops quickly and simultaneously, and the resulting error can be passed to all layers, the coefficients of which are updated effectively. This changes the inherent impression that sigmoid-liked neurons display slow convergences [4]. When the learning rate increases, the loss curve also increases because of the increase in the training errors.

**Figure 3:**
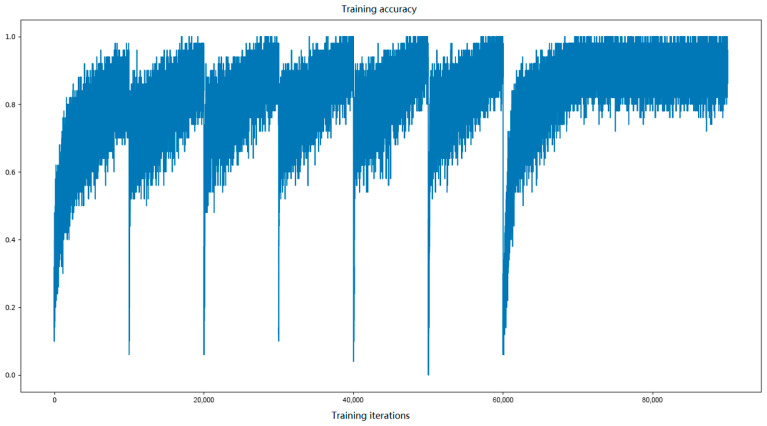
Training accuracy after each iteration.

Figure 3 shows the plot of training accuracy. As observed, the curve changes over a wide range, even during the same cycle. This is because each batch is selected randomly from the training dataset. When hyper coefficients are constant at the end of traning, the training accuracy is almost within the range of 80–100%.

**Figure 4:**
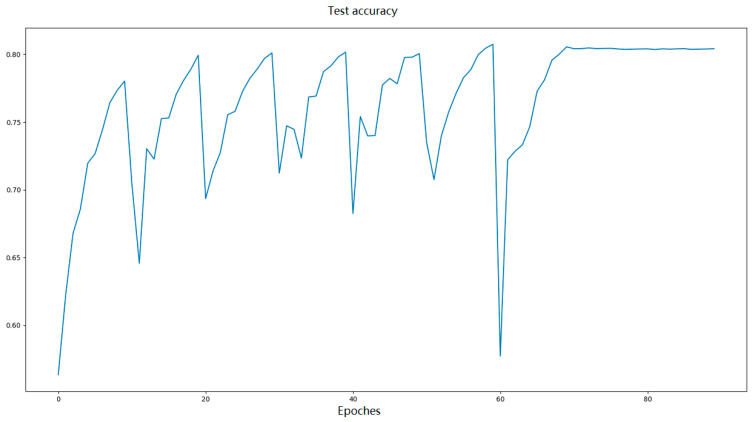
Test accuracy after each epoch of learning.

Figure 4 shows that the proposed algorithm reaches a 25% training error rate after the training of approximately 10 epochs, and the highest test accuracy of each period increases gradually. After 50 periods of the learning rate, the model almost reaches its best accuracy. After 90 training cycles, the model could reach 80.5% test accuracy. During the last 20 epochs, the learning rate is fixed, and the test accuracy was observed to fluctuate within a very shallow range, neither decreasing as usual because of overfitting nor increasing suddenly as in Resnet [5]. This proves that the use of tanh activation can make the model more stable, making the valid dataset unnecessary.

To explore whether model 1 lacks global information that leads to its low accuracy, model 2, which consisted of only 4 convolutional layers with more filters and 6 fully connected layers, was used for the same classification task. The simulation shows it nearly had the same accuracy as model 1. However, when we employed a simple data augmentation form, random horizontal reflections and random extracting 32 × 32 patches from the padded images [3], model 2 reached 84.19% accuracy but model 1 only increased to 82.61%.

To compare the performance of the models, applying tanh and ReLU actives as fairly as possible, we summarized the accuracies of several early algorithms in Table 2. The algorithms do not show good performance if no advanced methods are introduced in the training, even for ResNet 44.

To test the effect of noise on the trained model, two kinds of noises, namely with Gaussian and uniform distributions, were added to the test dataset:

(21) $$x^{\prime }=\hat{x}+noise\left\{\begin{array}{l} noise∼N(0,\sigma^{2})\\ noise∼U(-\sigma ,\;\sigma ) \end{array}\right.$$

where $\hat{x}$ denotes the normalized data.

We generated ten noises with the same distribution for each level and added them to the test dataset. The ten noisy datasets were fed into the model individually, and the average accuracy of the model was computed, as summarized in Table 3. According to the simulation, the model is more severely affected by Gaussian noise than by uniform noise. This may be because Gaussian noise has more peaks than uniform noise at the same σ.

### 4.2. CIFAR-100 Classification

To test whether our algorithm is suitable for a larger dataset, we increased the width of the ten used layers in the network to build model 3, which comprises five convolutional layers and five fully connect layers, as listed in Table 4.

To enhance the model, we set the cosine learning rate to increase at the first half of a period, which proved beneficial in warming up the model [29]. After 160 epochs of learning, the model reaches 61.23% test accuracy with simple data augmentation. Seemingly, the performance is not sufficient because of fewer layers and parameters, but the main reason lies in the limitation of the algorithm, proved in Section 5.

### 4.3. Target Classification of Unmanned-Aerial Vehicles

The Real Doppler RAD-DAR (RDRD) dataset [23,24] comprises three classes of Doppler-range images: cars, people, and drones. We randomly divided the data into three parts, namely the training set (70%), validation set (15%), and testing set (15%). Three images in sequence were used as one with three channels. The classification network and its parameters are the same as model 2 listed in Table 5. The table also compares the performances of different algorithms.

The proposed approach cannot outperform existing techniques in terms of accuracy, but given its minimal depth, minimal number of convolutional kernels, and minimal complexity, it should be able to perform on par with existing techniques. Furthermore, smaller networks can substitute the last fully connected layers, which means that network scale can be further decreased without sacrificing performance.

## 5. Analysis of the Model Classification Using an Explainable Approach

Applying an explainable method to analyze a deep learning network can help us understand why the model makes a particular prediction [30]. This paper utilizes a SHAP-based explainable method we developed (it will be presented in our future paper) to show how model 1 works. The core principle is to calculate the contribution of each pixel to the model output classification, of which the most important pixels are set to 1, and the least important ones are set to 0.

**Figure 5:**
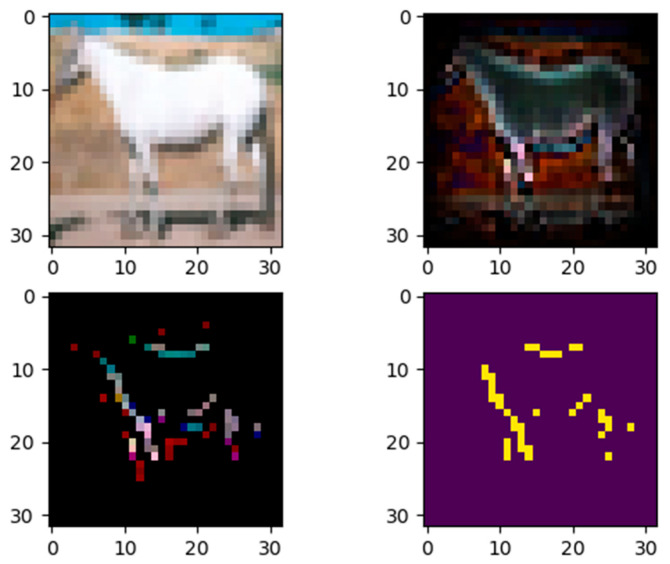
**Top left**: input image. **Top right**: the quantitative importance of each pixel. **Bottom left**: the importance map of top-right image where only pixels of importance ≥ 0.3 are kept, while other points are set to 0. **Bottom right**: binarization of bottom-left image after it was converted to gray image.

**Figure 6:**
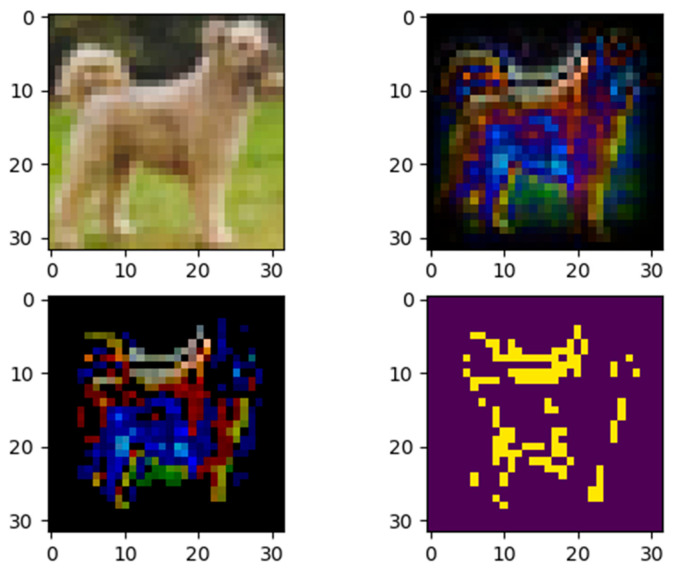
The model mistakenly classified the dog image into horses. **Top left**: input image. **Top right**: the quantitative importance of each pixel. **Bottom left**: the pixels of the gray image of top right with importance ≥ 0.3. **Bottom right**: binarization of the bottom-left image.

The next two examples input two images of different classes from the test data set to model 1 and calculate their contributions. For the first one, a horse picture was inputted, and the model outputted the correct label, as shown in Figure 5. However, in Figure 6, the second example shows that when inputting a dog image into the model, it outputted the wrong label, horse.

The input image (left) and the quantitative contribution image (RGB) of each pixel (right) are the two top images in the figure. The horse’s outline is clearly visible in the significance diagram, and the map’s black boundaries show that the margins have no bearing on the categorization outcomes. This is because convolution employs fewer border pixels and concentrates more on the image’s center. To obtain the bottom-left pixel, the pixels whose relevance is more than or equal to 0.3 are kept and the remaining points are set to 0. After that, the bottom-left map’s three channels (RGB) are averaged to obtain the important gray image and binarize it to obtain the image on the bottom right. The significance of internal information in the hose’s body is very low, as seen in the bottom two graphs, suggesting that the model did not successfully use them for categorization. This further suggests that the model ignores the image’s low frequency information in favor of concentrating more on the edge or high frequency.

Figure 6 is used to investigate the reasons behind the model’s inaccurate assessment. The model recognized a dog as a horse when it received a photo of one. The contribution map demonstrates that the model ignores other highly distinguishing traits, such as the head and tail, in favor of contours as a reliable classification criterion. The head features are conspicuously absent from the significance map, presumably because of convolution’s insensitivity to margin information. Furthermore, the feature map (top right and bottom left) indicates that the model gives greater attention to the blue channel.

**Figure 7:**
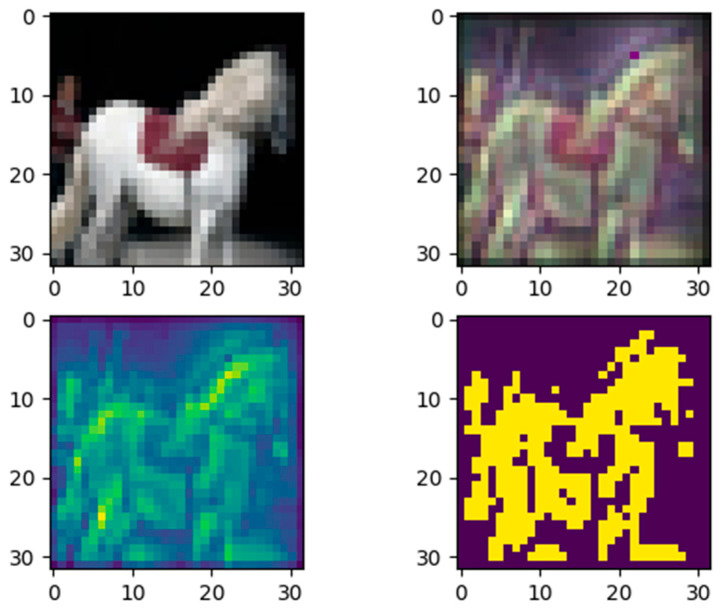
The model using ReLU activation made an correct classification. **Top left**: input image. **Top right**: the quantitative importance of each pixel. **Bottom left**: the pixels of the gray image of top right with importance ≥ 0.3. **Bottom right**: binarization of the bottom-left image.

We also designed and trained a classification model (achieved 76.7% accuracy) with the same structure as model 1 but using the ReLU activation function. Then, by applying the proposed explainable method, we generated a quantitative contribution map for each pixel of the input image in Figure 7. Unlike Figure 5 and Figure 6, the subplot bottom right shows that the model focuses on most of the information about the horse in the picture.

In addition to our explainable method, Selvaraju et al. [30] analyzed classical CNN and ResNet with ReLU using a visual explanation method, Grad-CAM; the generated heat images demonstrate that the networks did not significantly ignore the pixels at the image margins and instead looked at the distinctive internal features of targets and used those features for classification, rather than focusing on their contour.

The primary cause of the discrepancies in outcomes between the suggested algorithm and the ReLU-based CNN and ResNet is that, in our training procedure, some gradients with small amplitudes will vanish if they cannot be multiplied by the future gradients with amplitudes bigger than 1. The model will become more likely to concentrate on the edges of the image and other features with higher gradients.

Consequently, deep learning networks use them concurrently as activations due to their complementary advantages, which makes this a potentially workable method of enhancing learning capacity.

## 6. Conclusions

To ensure that the gradient may be effectively propagated back to the lowest layer, our method enlarges the gradient of some layers to be greater than 1; this prevents the condition of the vanishing gradient. Using this strategy, we trained a deep learning network with tanh activation, which can rapidly reach convergence. We employed interpretability approaches to determine the information that the model is most concerned with, in order to obtain insight into the algorithm’s working mechanism. We then studied the distinctions between this method and other prominent methods currently in use, finding that they concentrate on various features. This suggests that our approach could find use in picture segmentation tasks. Additionally, integrating two activation functions into a deep learning network is a viable way to enhance network capabilities.

## Figures and Tables

**Table 1:** Model 1 and 2 structures and hyper-parameters.

Layer	Model 1	Model 2
Conv size/stride	3 × 3 × 32/1 Padding = ‘SAME’	3 × 3 × 40/1 Padding = ‘SAME’
Conv size/stride	3 × 3 × 32/1 Padding = ‘SAME’	3 × 3 × 40/1 Padding = ‘SAME’
Conv size/stride	3 × 3 × 32/1 Padding = ‘SAME’	
Pool size/stride	2 × 2/2 Max Out	2 × 2/2 Max Out
Conv size/stride	3 × 3 × 32/1 Padding = ‘SAME’	3 × 3 × 52/1 Padding = ‘VALID’
Conv size/stride	3 × 3 × 32/1 Padding = ‘SAME’	3 × 3 × 56/1 Padding = ‘VALID’
Conv size/stride	3 × 3 × 32/1 Padding = ‘SAME’	
Pool size/stride	2 × 2/2 Max Out	2 × 2/2 Max Out
Conv size/stride	3 × 3 × 40/1 Padding = ‘SAME’	
Conv size/stride	3 × 3 × 40/1 Padding = ‘SAME’	
Pool size/stride	2 × 2/2 Max Out	
Dense	128 + 10	1024 + 512 + 384 + 124 + 64 + 10
Loss	Softmax + cross-entropy	
Cosine Learning rate	If epochs ≤ 70:	If epochs ≤ 80:
	Lr_max = 0.1 Lr_min = 0.001	Lr_max = 0.1 Lr_min = 0.0005
	Else:	Else:
	Lr = 0.0001	Lr = 0.0005
The period of cosine	T = 10 epochs	T = 20 epochs
L2 regularization	0.003	0.005

**Table 2:** Test accuracies of different algorithms without data augmentation.

Model	Algorithm/Optimizer	Test Accuracy
	GD	79.77%
AlexNet [21]	Adam	79.67%
	EVGO	80.92%
ResNet44	w/flood [28]	75.52%
Model 1 + tanh	PN/SGD	80.50%
Model 2 + tanh	PN/SGD	80.20%

The accuracies with underline _ are the results of our algorithms.

**Table 3:** Accuracies on different noise levels for model 2.

σ	0	0.02	0.04	0.06	0.08	0.1
Gaussian	84.19%	83%	75.9%	61.5%	44.9%	32.9%
Uniform	84.19%	83.9%	81.8%	75.8%	65.4%	53.3%

**Table 4:** Model 3 structure and its parameters.

Conv size/stride	3 × 3 × 80/1	Padding = ‘SAME’
Pool size/stride	2 × 2/2	Max out
Conv size/stride	3 × 3 × 96/1	Padding = ‘SAME’
Pool size/stride	2 × 2/2	Max out
Conv size/stride	3 × 3 × 112/1	Padding = ‘SAME’
Conv size/stride	3 × 3 × 136/1	Padding = ‘SAME’
Conv size/stride	3 × 3 × 168/1	Padding = ‘VALID’
Pool size/stride	2 × 2/2	Max out
Dense	3072 + 1024 + 512 + 320 + 100	
loss	Softmax + cross-entropy	
Cosine Learning rate	Lr_max = 0.1 Lr_min = 0.0001	
The period of cosine	T = 30 epochs	
L2 regularization	0.001	

**Table 5:** Accuracies of different models.

Model	Average Accuracy
NasNetMobile [23]	0.9769
MobileNetV2 [23]	0.9894
DopplerNet [23]	0.9948
Inception–Residual model [24]	0.985
Our Model 2	0.975

The accuracy with underline _ is the result of our algorithm.

## Data Availability

The datasets can be obtained from the website: www.cs.toronto.edu/~kriz/cifar.html, (accessed on 25 December 2025).
